# A gel-filled hood technique to maintain a clear visual field during underwater endoscopic submucosal dissection

**DOI:** 10.1055/a-2865-2650

**Published:** 2026-06-01

**Authors:** Nobutoshi Tsukuda, Shunsuke Yoshii, Tomoki Michida, Ryu Ishihara

**Affiliations:** 1 Department of Gastrointestinal Oncology 53312 Osaka International Cancer InstituteOsakaJapan

Underwater endoscopic submucosal dissection (UESD 1 ) is a useful technique; however, endoscopic visualization can easily be impaired by bleeding or bubbles. Although several instruments and techniques have been developed to address this issue 2 3 , they are often complex or costly. Here, we propose a simple gel-filled hood technique to maintain a clear visual field during UESD with saline immersion ( Video 1 ).

Underwater endoscopic submucosal dissection for a rectal tumor using the gel-filled hood technique.Video 1

**Fig. 1:**
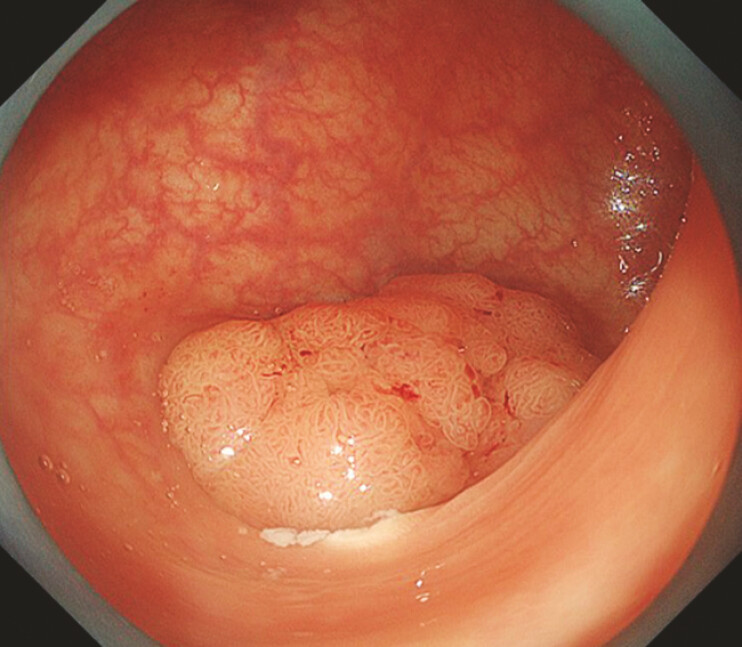
An endoscopic image of a rectal tumor.

**Fig. 2:**
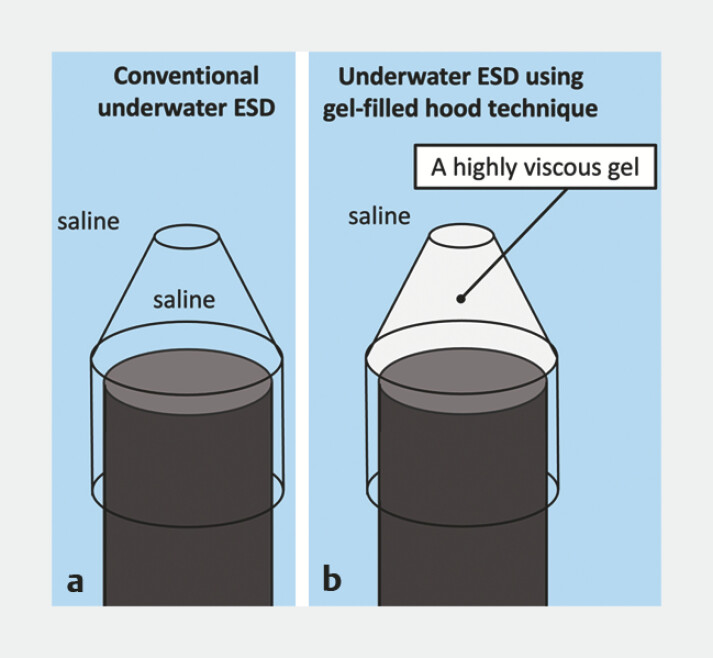
Schematic illustration of conventional UESD and the gel-filled hood technique. **a** The conventional UESD: The entire hood, both inside and outside, is immersed in saline. **b** The gel-filled hood technique: The tapered hood is filled with a highly viscous gel inside, while the outer space is filled with normal saline. UESD, underwater endoscopic submucosal dissection.

**Fig. 3:**
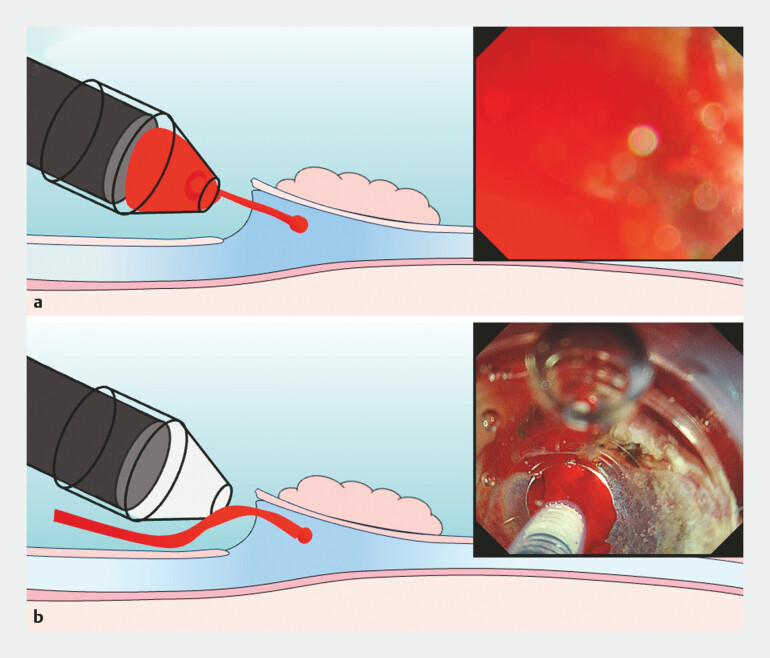
Comparison of schematic illustrations and endoscopic images showing the effect of blood ingress on the visual field during conventional UESD and the gel-filled hood technique. **a** Conventional UESD (a reference endoscopic image from a different case): Blood enters the hood and obscures the visual field, making it difficult to identify the bleeding point. The endoscopic image from a separate case shows visual obstruction due to the blood. **b** The gel-filled hood technique: The highly viscous gel inside the hood prevents blood from entering, maintaining a clear visual field. The endoscopic image demonstrates unobstructed visibility despite the active bleeding. UESD, underwater endoscopic submucosal dissection.

A 73-year-old woman was referred to our center for endoscopic resection of a 20 mm rectal laterally spreading tumor ( Fig. 1 ). UESD with saline immersion was performed using a two-channel endoscope (GIF-2TQ260M; Olympus, Tokyo, Japan) and a tapered transparent hood (CAST hood; TOP, Tokyo, Japan). During the UESD procedure, endoscopic visualization can be impaired by intraoperative bleeding and bubbles. We therefore filled the tapered hood with a highly viscous lubricating gel not intended for gel immersion endoscopy. The gel could be retained inside the hood even under saline immersion owing to the small opening of the tapered hood and high viscosity of the gel ( Fig. 2 ), thereby preventing blood and bubbles from entering the hood ( Fig. 3 ). Using this gel-filled hood technique, complete en bloc resection was achieved without any adverse events.

Bubbles generated by the repeated application of electric current can interfere with endoscopic visualization. These bubbles tend to accumulate within the hood, especially when a tapered hood is used, and are often difficult to remove. Similarly, blood entering the hood can obscure the visual field, and make it difficult to identify the bleeding point under water. Our novel technique was designed to prevent the ingress of bubbles and blood into the hood, thereby maintaining an unobstructed and clear visual field during the UESD procedure. The gel-filled hood technique requires a small amount of gel only inside the hood, providing a simple and cost-effective approach for maintaining a clear visualization.

Endoscopy_UCTN_Code_TTT_1AQ_2AD_3AD
